# Potential role of the comprehensive tooth extraction procedure in preventing medication related osteonecrosis of the jaw (MRONJ): a prospective cohort study

**DOI:** 10.1186/s13023-025-04138-9

**Published:** 2025-12-09

**Authors:** Yi Wang, Yu Zhang, Dengke Li, Wuyang Zhang, Shuming Wang, Xueni Zheng, Yuan Li, Tiange Deng, Chunlin Zong, Lei Tian, Ping Liu, Yang Xue

**Affiliations:** 1 State Key Laboratory of Oral and Maxillofacial Reconstruction and Regeneration, Clinical Research Center for Oral Diseases, Department of Oral and Maxillofacial Surgery, School of Stomatology, National Clinical Research Center for Oral Diseases, The Fourth Military Medical University, Xi’an, Shaanxi China; 2 Department of Stomatology, Air Force Hospital of Southern Theater Command, Guangzhou, China

**Keywords:** Medication-related osteonecrosis of the jaw (MRONJ), Concentrated growth factor (CGF), Tooth extraction, Perioperative care

## Abstract

**Background:**

This article aims to elucidate the potential role of a comprehensive tooth extraction procedure in preventing mdication-related osteonecrosis of the jaw (MRONJ) through a prospective cohort study. By systematically assessing clinical outcomes following this procedure, the study seeks to provide evidence regarding its effectiveness in MRONJ prevention, thereby contributing to improved clinical guidelines and patient care related to dental extractions in at-risk populations.

**Methods:**

Patients using anti-resorptive agents (ARAs) who required extraction of at least one tooth were included in the study. Patients’ medical history, medication history, and intraoral dental conditions were documented, and CBCT scans were performed. Following a standardized treatment protocol, patients received professional oral cleaning and antibiotics preoperatively. During surgery, minimally invasive extraction and concentrated growth factor (CGF) filling were performed with meticulous suturing whenever possible. Postoperatively, mouthwash was used within one month. Follow-up visits were scheduled at 10, 30, and 90 days to monitor and analyze MRONJ incidence and surgical outcomes.

**Results:**

A total of 101 patients were included in the study, with 20 receiving oral ARAs for osteoporosis, 57 receiving intravenous ARAs for osteoporosis, 13 undergoing combination therapy for osteoporosis, and 11 using ARAs for malignancy. Zoledronic acid and denosumab were the most commonly used drugs. Increased bone density was observed on preoperative CBCT in 26 patients, and on postoperative CBCT at 90 days in 31 patients. In total, 248 teeth were extracted, mostly due to severe defects; periapical periodontitis and periodontitis were also major reasons for extraction. Most patients could not achieve complete and tight suturing. All patients remained free of MRONJ during the 90‑day postoperative period, with complete mucosal healing in every case.

**Conclusion:**

This prospective cohort study provides evidence that implementing an effective and rational treatment protocol during tooth extractions significantly benefits high-risk MRONJ patients. Adherence to such protocols minimizes the risk of postoperative infection, fosters improved healing of extraction sites, and maximizes the prevention of MRONJ.

**Clinical trial number:**

Not applicable.

## Introduction

Medication-related osteonecrosis of the jaw (MRONJ) is a metabolic bone disorder characterized by necrosis of the jawbone. It predominantly develops in patients treated with anti-resorptive agents (ARAs), commonly prescribed for the management of osteoporosis and bone metastases linked to malignancy [1, 2]. According to the American Association of Oral and Maxillofacial Surgeons (AAOMS), MRONJ is defined by three criteria: the presence of exposed necrotic bone persisting for more than eight weeks, a history of current or previous exposure to antiresorptive or antiangiogenic agents, and no prior radiation therapy or malignant disease involving the jaws [3]. MRONJ can manifest through various symptoms, including delayed wound healing, localized gingival erythema, swelling, pain, and recurrent purulent discharge. Additionally, patients may experience jawbone necrosis and, in more severe cases, exposure of necrotic bone and pathological fractures. These complications can significantly diminish a patient’s quality of life [4]. Current management strategies emphasize delayed surgical interventions, such as debridement or segmental resection with reconstruction. However, these approaches are hampered by the difficulty in precisely identifying the borders of necrotic bone during surgery. This often leads to recurrence, persistent functional impairment, and an increased risk for patients with significant comorbidities [5]. Consequently, the adoption of preventive, measures to suppress infection and promote local healing are of critical clinical importance, given their potential to reduce morbidity, prevent disease progression, and preserve patients’ overall well-being. MRONJ demonstrates a variable incidence depending on the patient population and dosing regimen, ranging from 0.043% to 0.215% among osteoporosis patients receiving low-dose ARAs for fracture prevention, and increasing substantially to 0.644%–2.731% in individuals with solid tumor bone metastases or multiple myeloma managed with high-dose therapies [6]. Tooth extraction emerges as the most prevalent local precipitating factor, implicated in approximately 61% of MRONJ cases [7]; nonetheless, other dental interventions such as bone biopsies, crown lengthening, bone surgeries, implant placement, and chronic inflammatory conditions like untreated periodontitis can also provoke MRONJ by inducing trauma and creating a need for bone remodeling. This physiological process is impaired by ARAs [8–10]. The occurrence of MRONJ is further linked to the inhibition of osteoclast activity, which diminishes the capacity for bone repair, and the suppression of angiogenesis, resulting in reduced blood supply to affected regions [11]. Moreover, the oral cavity’s complex microbial environment and the disruption of the mucosal barrier during invasive procedures can facilitate infection of the underlying bone tissue [12, 13]. Despite these recognized risk factors and mechanisms, the precise pathogenesis of MRONJ remains incompletely understood.

If tooth extraction is necessary in patients at risk for MRONJ, several measures can effectively reduce the incidence of this complication. Improving the oral microbial environment through professional cleaning and strict oral hygiene prior to surgery prepares the tissues for optimal healing [14, 15]. Employing gentle surgical techniques to minimize trauma, alongside perioperative administration of antimicrobial agents, further lowers the risk of infection and promotes recovery. Together, these preventative strategies provide a practical framework for safely managing tooth extractions in susceptible individuals, thereby enhancing patient outcomes and procedural safety.

This article aims to elucidate the potential role of a comprehensive tooth extraction procedure in preventing MRONJ through a prospective cohort study of 103 patients. By systematically assessing clinical outcomes following this procedure, the study seeks to provide evidence regarding its effectiveness in MRONJ prevention, thereby contributing to improved clinical guidelines and patient care related to dental extractions in at-risk populations.

## Methods

### Study design

This prospective cohort study received approval from the Human Subjects Ethics Board of the Fourth Military Medical University, the Third Affiliated Hospital (Decision number: KQ-YJ-2024-086), and was conducted in compliance with the principles outlined in the Declaration of Helsinki (1975), as revised in 2013.

To be eligible for inclusion, patients were required to have a history of ARAs use and to necessitate extraction of one or more teeth. Exclusion criteria comprised a history of radiation therapy to the jaws, a history of MRONJ, presence of metastatic disease involving the jaws, inability to tolerate surgical procedures due to physical condition, or failure to complete a three-month postoperative follow-up. All patients sign an informed surgical consent form prior to the procedure.

### Clinical data collection

Clinical data collected for each patient included name, age, indication for ARAs use, medication history, dosage and frequency of administration, indication for tooth extraction, and history of other systemic diseases. Cone-beam computed tomography (CBCT) scans were performed preoperatively and at 90 days postoperatively. Clinical intraoral examinations were conducted at 10, 30, and 90 days postoperatively to monitor for the development of MRONJ. The diagnosis of MRONJ was established based on the criteria outlined by the AAOMS guidelines [16].

### The comprehensive tooth extraction procedure

**Fig. 1:**
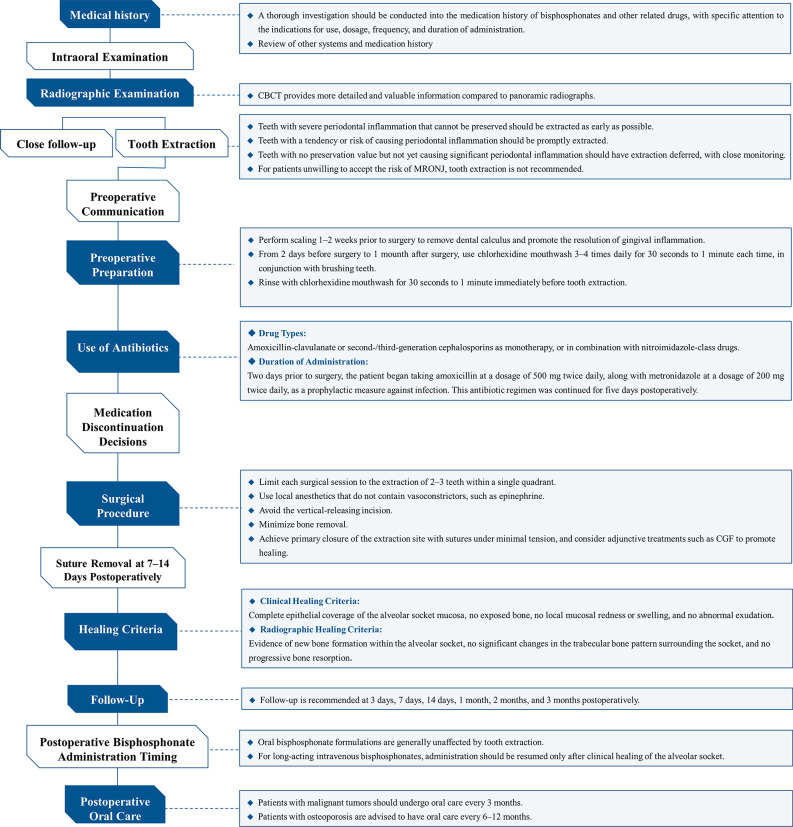
The comprehensive tooth extraction procedure for patients using ARAs medication

**Fig. 2:**
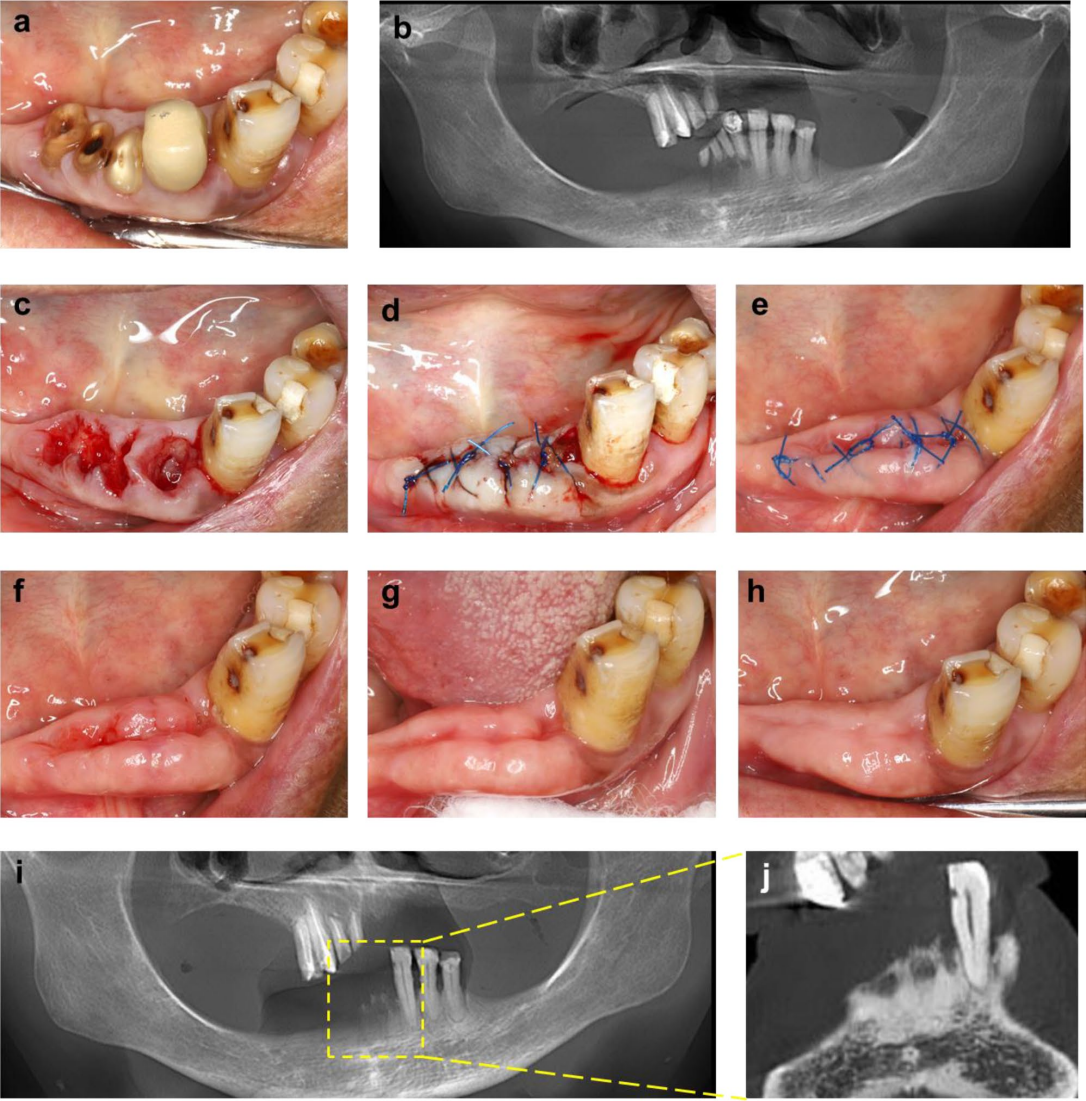
Clinical tooth extraction surgical steps and mucosal healing conditions postoperatively. (**a**) Preoperative intraoral condition. (**b**) Preoperative CBCT image of the patient. (**c**) Immediate intraoral condition after tooth extraction. (**d**) The extraction wound was sutured with 4 − 0 non-absorbable sutures. (**e**) Healing condition of the extraction site 10 days postoperatively. (**f**) Suture removal. (**g**) Healing condition of the extraction site 30 days postoperatively. (**h**) Healing condition of the extraction site 90 days postoperatively. (**i**)-(**j**) CBCT images of the patient at 90 days postoperatively

The comprehensive tooth extraction protocol is depicted in Fig. 1, with a representative case presented in Fig. 2. Prior to extraction, patients undergo a thorough medical history evaluation and CBCT scan. One week before surgery, full-mouth dental scaling is performed to reduce oral plaque levels. Two days prior to the surgical procedure, the patient initiated prophylactic antibiotic therapy, consisting of amoxicillin 500 mg administered twice daily and metronidazole 200 mg administered twice daily, to reduce the risk of postoperative infection. With the patient’s informed consent, 20 mL of venous blood is collected preoperatively to prepare concentrated growth factor (CGF). All extractions are performed by an experienced specialist. Local anesthesia is achieved with 4% articaine. Extraction instruments are selected according to the specific dental condition to ensure minimally invasive tooth removal. Granulation tissue and any sharp bone spicules are meticulously debrided. The extraction socket is irrigated with saline, after which a piece of CGF gel is placed into the socket, and an additional piece is compressed into a membrane to cover the extraction site. Primary closure of the wound is typically achieved using 4 − 0 monofilament, nonabsorbable polypropylene sutures; if this is not feasible, wound edges are approximated as closely as possible. Postoperative management includes a five-day course of antibiotics to prevent infection. For patients with a penicillin allergy, we prescribe clarithromycin for infection prophylaxis. Patients are advised to rinse with chlorhexidine solution, tinidazole solution, or povidone‑iodine mouthwash three times daily for one month to reduce the oral bacterial load. Sutures are removed 10 days after surgery, and follow-up assessments are conducted at 30 and 90 days to monitor socket healing. ARAs may be resumed one month postoperatively if indicated.

To ensure strict compliance with the predefined preventive protocol, we implemented systematic adherence-monitoring measures. Preoperative cleaning was verified through medical record review; preoperative antibiotic use was confirmed on the day of surgery via patient interview and prescription reconciliation. All surgical steps strictly followed standardized procedures and were documented in the operative record. For postoperative mouthwash use, in addition to detailed oral and written instructions, adherence was reinforced and verified through telephone follow-ups on postoperative days 1, 3, and 7, as well as during subsequent clinic visits.

### Statistical analysis

Descriptive statistics were utilized to summarize the characteristics of the patient population. All statistical analyses were conducted using SPSS software, version 29.0 (IBM, Armonk, New York, USA). Patients were categorized based on the route of administration and the therapeutic purpose of ARAs as follows: Group A, oral ARAs for osteoporosis; Group B, intravenous ARAs for osteoporosis; Group C, combination therapy for osteoporosis; and Group D, intravenous ARAs for cancer treatment. If the data in the tables are normally distributed, they are presented as mean ± standard deviation; otherwise, as median (interquartile range). The Kruskal-Wallis test was employed to evaluate differences in mucosal healing status among these groups.

## Results

### Demographic data and medication information

**Fig. 3:**
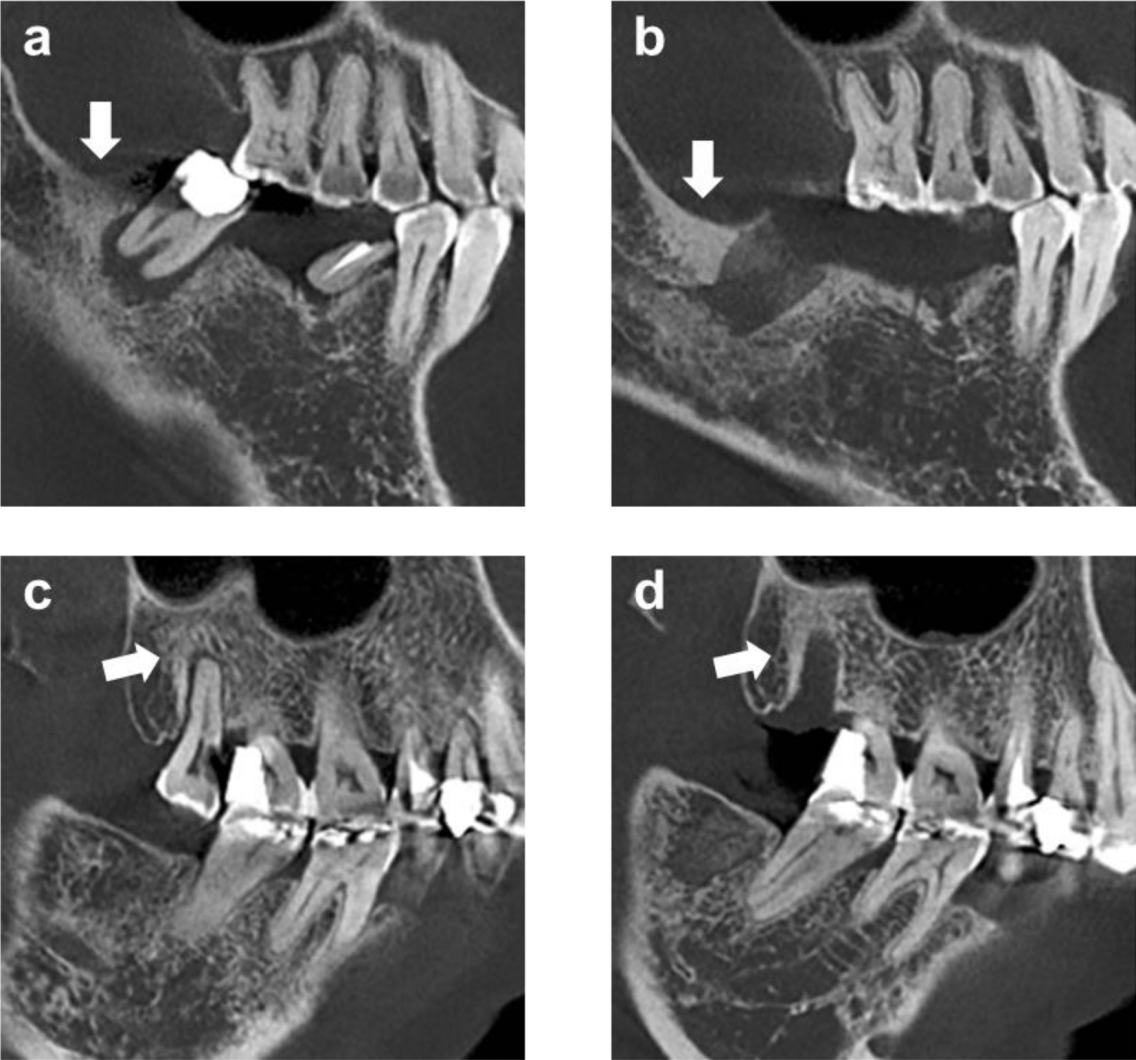
Changes in bone density observed in the patient’s CBCT image. (**a**) Pre-extraction imaging. (**b**) Post-extraction imaging reveals an increase in bone density at the extraction site. (**c**) Pre-extraction imaging reveals an increase in bone density at the extraction site. (**d**) Increased bone density showed no significant change postoperatively

A total of 101 patients who received ARAs and underwent tooth extraction at the Third Affiliated Hospital of the Fourth Military Medical University between October 2013 and October 2024 were included in this study, according to specified inclusion and exclusion criteria. Table 1 summarizes baseline medication characteristics for patients with osteoporosis (*n* = 90) and cancer (*n* = 11). The osteoporosis group had an age range of 45–95 years (mean ± SD: 67.76 ± 9.70 ), whereas patients in the cancer group were relatively younger, with an age range of 36–79 years (mean ± SD: 57.18 ± 13.62).In the osteoporosis cohort, most patients were aged 61–80 years (66.67%), with 24.44% aged ≤ 60 and 8.89% aged ≥ 81. In contrast, the cancer cohort was predominantly ≤ 60 years (63.64%), with 36.36% aged 61–80 and no patients ≥ 81 years. The cohorts contributed 222 and 26 teeth, respectively. Zoledronic acid was the most commonly used agent in both cohorts (osteoporosis: 41.11%; cancer: 72.73%). Ibandronic acid (16.67%), alendronic acid (27.78%), and denosumab (27.78%) were used only in the osteoporosis group. The duration of medication use ranged from 4 to 504 weeks in the osteoporosis cohort and from 1 to 192 weeks in the cancer cohort. Reported drug holidays ranged from 12 to 96 weeks and from 4 to 24 weeks, respectively. Among all the patients, 20 patients took oral medications for osteoporosis, 57 received injectable medications for osteoporosis, 13 underwent combination therapy for osteoporosis, and 11 were treated for cancers via ARAs injections (Table 2). The majority of patients (63.37%) were aged 61–80 years, though most (63.63%) cancer-treatment patients were under 60 years. Alendronate (95.00%) was the predominant oral agent, whereas zoledronic acid (47.37%) was most frequently administered via injection. Most patients underwent a single tooth extraction, while a minority required multiple procedures. Another noteworthy observation is the increase in bone density detected through preoperative CBCT imaging in 26 patients, which was documented in 31 patients three months after tooth extraction (Fig. 3). More detailed patient characteristics are listed in Table 2.

**Table 1:** Baseline information on patient medication use

Characteristic	Osteoporosis(*n* = 90)	Cancer(*n* = 11)
Age (years old), No. (%)		
≤ 60	22(24.44%)	7(63.64%)
61–80	60(66.67%)	4(36.36%)
≥ 81	8(8.89%)	0
Age range (years old)	45–95(67.76 ± 9.70)	36–79(57.18 ± 13.62)
**Sex**,** No. (%)**		
Male	9(10.00%)	2(18.18%)
Female	81(90.00%)	9(81.82%)
**Tooth number**,** No.**	222	26
**Medication**,** No. (%)**		
Zoledronic acid	37(41.11%)	8(72.73%)
Ibandronic acid	15(16.67%)	0
Alendronic acid	25(27.78%)	0
Denosumab	25(27.78%)	0
Others	6^a^(6.67%)	3^b^(27.27%)
**Duration of medication use (Weeks)**	4-504(131 [73, 209]) ^c^	1-192(96.09 ± 52.74)
**Drug Holiday (Weeks)**	12–96(53.56 ± 25.94)	4–24(12.45 ± 6.35)

a: Risedronate; Elcatonin, Clodronate; Carbocalcitonin; Teriparatide

b: Bevacizumab, Icotinib, Incadronate

c: Data do not follow a normal distribution; use the median

**Table 2:** Demographic data and medication information of the included patients

Characteristic	Group A(*N* = 20)	Group B (*N* = 57)	Group C (*N* = 13)	Group D (*N* = 11)	
Age groups, No. (%)					
≤ 60 years old	6(30.00)	13(22.80)	3(23.08)	7(63.63)	
61–80 years old	12(60.00)	39(68.42)	9(69.23)	4(36.37)	
≥ 81 years old	2(10.00)	5(8.77)	1(7.69)	0(0.00)	
**Sex**,** No. (%)**					
Male	3(15.00)	6(10.53)	0(0.00)	2(18.18)	
Female	17(85.00)	51(89.47%)	13(100.00)	9(81.82)	
**Medication**,** No. (%)**					
Zoledronic acid	0(0.00)	27(47.37)	10(76.92)	8(72.73)	
Ibandronic acid	0(0.00)	7(12.28)	8(61.64)	0(0.00)	
Alendronic acid	19(95.00)	0(0.00)	6(46.15)	0(0.00)	
Denosumab	0(0.00)	21(36.84)	4(30.77)	0(0.00)	
Others	1^a^(5.00)	2 ^b^(3.51)	3 ^c^(23.08)	3 ^d^(27.27)	
**Combined Dexamethasone**,** No. (%)**					
Yes	1(5.00)	1(1.75)	0(0.00)	0(0.00)	
No	19(95.00)	56(98.25)	13(100.00)	11(100.00)	
**Diabetes history**,** No. (%)**					
Yes	1(5.00)	2(3.51)	0(0.00)	0(0.00)	
No	19(95.00)	55(96.49)	13(100.00)	11(100.00)	
**Times of tooth extraction**,** No. (%)**					
1	16(80.00)	39(68.42)	12(92.31)	6(54.55)	
2	4(20.00)	10(17.54)	1(7.69)	4(36.36)	
≥ 3	0(0.00)	8(14.04)	0(0.00)	1(9.09)	
**Number of teeth extracted per visit (Mean ± SD)**					
	1.48 ± 0.70	1.65 ± 0.87	1.44 ± 0.59	1.48 ± 0.66	
**Types of mouthwash after the surgery**,** No. (%)**					
Povidone-iodine	12(60.00)	32(56.14)	6(46.15)	11(100.00)	
Chlorhexidine	3(15.00)	13(22.81)	7(53.85)	0(0.00)	
Tinidazole	5(25.00)	12(21.05)	0(0.00)	0(0.00)	
**Increased bone density at the extraction site before surgery**,** No. (%)**					
Yes	5(25.00)	10(17.54)	5(38.46)	6(54.55)	
No	15(75.00)	47(82.46)	8(61.54)	5(45.45)	
**Increased bone density at the extraction site after surgery**,** No. (%)**					
Yes	5(25.00)	17(29.82)	3(23.08)	6(54.55)	
No	15(75.00)	40(70.18)	10(76.92)	5(45.45)	

a: Risedronate; b: Elcatonin, Clodronate; c: Carbocalcitonin, Teriparatide;

d: Bevacizumab, Icotinib, Incadronate

### Characteristics of the extracted teeth and MRONJ

A total of 248 teeth were removed across various treatment groups, with the highest number in the injectable osteoporosis therapy group (*n* = 164). Molars accounted for the majority of extractions, and the leading cause was irreparable residual crowns or roots (42.74%). Other significant indications included apical pathology (22.58%) and periodontitis (20.56%). Surgical procedures frequently involved flap elevation (38.31%) and bone removal with a handpiece (25.00%). CGF was utilized in socket filling for most extractions (98.79%), while a large proportion of sites (74.60%) were not tightly sutured postoperatively. Further surgical details are summarized in Table 3. None of the extraction sites developed MRONJ.

**Table 3:** Characteristics of the extracted teeth and MRONJ(tooth number = 248)

Characteristic	Group A(*N* = 37)	Group B (*N* = 164)	Group C (*N* = 21)	Group D (*N* = 26)	
Jaws, No. (%)					
Maxillary	14(37.84)	98(59.76)	11(52.38)	15(57.69)	
Mandibular	23(62.16)	66(40.24)	10(47.62)	11(42.31)	
**Tooth location**,** No. (%)**					
Anterior teeth	6(16.22)	42(25.61)	4(19.05)	5(19.23)	
Premolars	8(21.62)	35(21.34)	3(14.29)	8(30.77)	
Molars	23(62.16)	87(53.05)	14(66.67)	13(50.00)	
**Dental diagnosis**,** No. (%)**					
Unrestorable tooth	22(59.46)	67(40.85)	7(33.33)	10(38.46)	
Apical pathology	7(18.92)	36(21.95)	4(19.50)	9(34.62)	
Periodontal disease	4(10.81)	35(21.34)	7(33.33)	5(19.23)	
Pulpitis	1(2.70)	12(7.32)	3(14.29)	0(0.00)	
Fracture of tooth	2(5.41)	14(8.54)	0(0.00)	1(3.85)	
Unrestorable implant	1(2.70)	0(0.00)	0(0.00)	0(0.00)	
**Flap surgery**,** No. (%)**					
Yes	15(40.54)	61(37.20)	14(66.67)	5(19.23)	
No	22(59.46)	103(62.80)	7(33.33)	21(80.77)	
**Bone remove**,** No. (%)**					
Yes	4(10.81)	37(22.56)	7(33.33)	14(53.85)	
No	33(89.19)	127(77.44)	14(66.67)	12(46.15)	
**CGF filling**,** No. (%)**					
Yes	35(94.59)	164(100.00)	21(100.00)	25(96.15)	
No	2(5.41)	0(0.00)	0(0.00%)	1(3.85)	
**Tight suturing**,** No. (%)**					
Yes	6(16.22)	44(26.83)	3(14.29)	10(38.46%)	
No	31(83.78)	120(73.17)	18(85.71)	16(61.54%)	
**Postoperative MRONJ at the extraction site**,** No. (%)**					
Yes	0(0.00)	0(0.00)	0(0.00)	0(0.00)	
No	37(100.00)	164(100.00)	21(100.00)	26(100.00)	

### Oral mucosal healing status of the patients

At the time of suture removal, 10 days postoperatively, complete mucosal healing at the extraction sites was not observed in any patients. In group A, 17 extraction sites demonstrated basic mucosal healing. In Group B, 52 extraction sites exhibited basic healing. For patients in group C, only 4 extraction sites achieved basic mucosal healing. Notably, in group D, none of the extraction sites demonstrated satisfactory mucosal healing. Statistical analysis revealed a significant difference in the distribution of basic healing and complete non-healing among the groups (H = 17.131, *P* < 0.001, effect size = 0.263, 95% CI ≈ 0.144–0.382). Pairwise comparisons indicated that Group D differed significantly from both Group A and Group B.

At the 30-day postoperative follow-up, complete mucosal healing was observed in 10.81% of extraction sites within Group A. In Group B, 25 extraction sites achieved full mucosal healing. All extraction sites in group C exhibited basically mucosal healing. In the cancer therapy group, 15.38% of extraction sites achieved full mucosal healing. Intergroup comparisons demonstrated no significant difference in the number of fully healed cases among all groups (H = 10.035, *P* = 0.253).

**Fig. 4:**
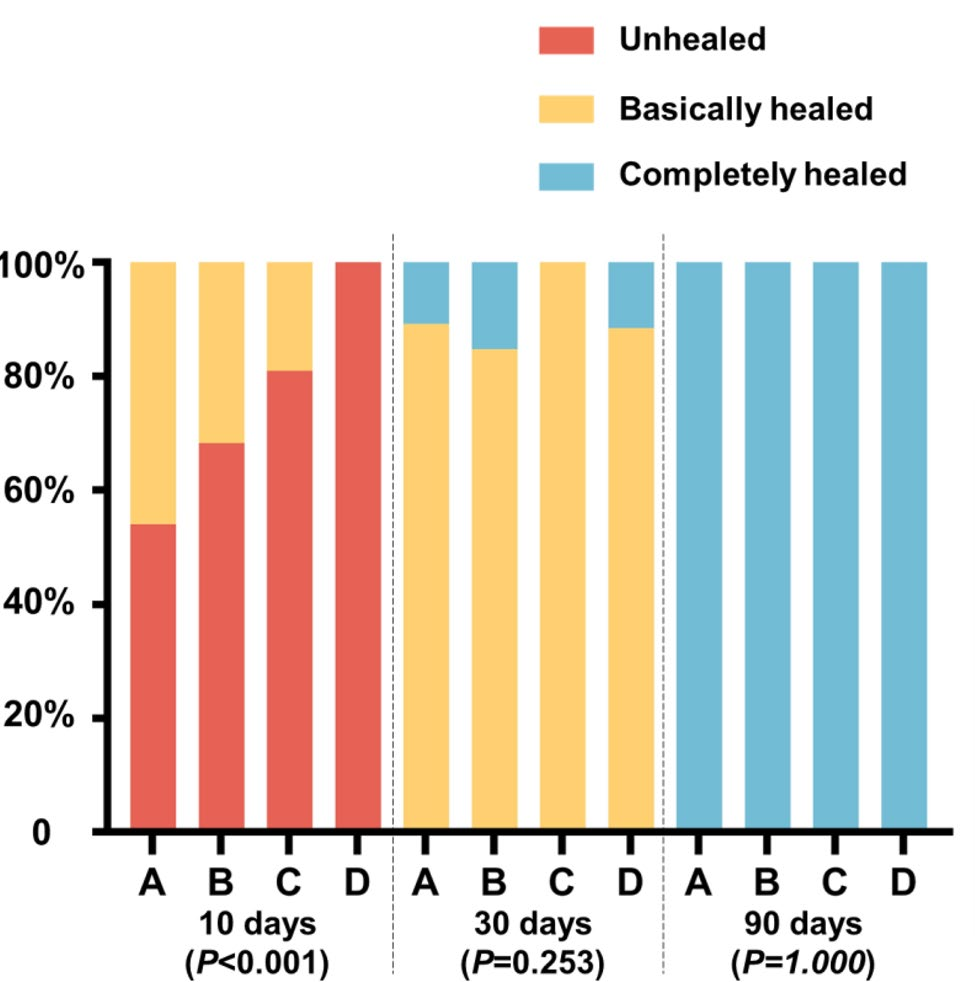
Mucosal healing condition of the patients. Group **A**: Oral ARAs for the treatment of osteoporosis. Group **B**: Intravenous ARAs for the treatment of osteoporosis. Group **C**: Combination therapy for the treatment of osteoporosis. Group **D**: Intravenous ARAs for the treatment of cancers

At the 90-day postoperative follow-up, all extraction sites had completely healed. Details regarding mucosal healing in individual patients are illustrated in Fig. 4. Notably, some cancer patients were able to continue anti-resorptive therapy without complication.

## Discussion

This prospective cohort study evaluated the efficacy of a comprehensive tooth extraction protocol in preventing MRONJ among 101 high-risk patients receiving ARAs. The results demonstrated that none of the 248 extraction sites developed MRONJ during the 90-day follow-up period, and all sites achieved complete mucosal healing by the final follow-up. These findings suggest that a structured perioperative management strategy—incorporating preoperative oral hygiene optimization, antibiotic prophylaxis, minimally invasive surgery, CGF application, and postoperative antimicrobial mouthwash—may significantly reduce the incidence of MRONJ following dental extractions.

The absence of MRONJ in our cohort is particularly noteworthy given the high-risk profile of the included patients. The incidence of MRONJ demonstrates significant variability, primarily influenced by factors such as medication type, dosage, and the route of administration, with reported rates ranging from 0.4% to 21% [17, 18]. The prevention of MRONJ, achieved by managing its etiological factors, is paramount for reducing patient morbidity [19, 20]. Recognized clinical risk factors for MRONJ include both patient- and treatment-related elements, with dental risk factors being particularly prominent; among these, tooth extraction stands out as the most significant risk, followed closely by periodontal disease and pre-existing oral infections, such as periapical conditions. Several studies have highlighted these associations. For instance, Beninati et al. observed that a majority of MRONJ cases were preceded by invasive dental procedures, mainly tooth extractions [21], while Tardast et al. found a similar prevalence of recent extractions and corticosteroid use among patients with MRONJ [22]. In this study, even among the 11 patients in Group D (malignancy cohort), who are considered at the highest risk, no cases of MRONJ occurred. However, during the same study period, two other patients who underwent tooth extraction in our department developed MRONJ after failing to disclose their medication history prior to the procedure, thereby precluding application of the comprehensive preventive protocol. The first case involved a 91-year-old male with a history of prostate cancer who had been receiving monthly intravenous zoledronic acid for five years and presented for extraction of two fractured right mandibular molars. The second case was a 45-year-old female with a prior history of surgical treatment for an inflammatory myofibroblastic tumor of the right maxilla in 2012, who received four intravenous injections of zoledronic acid between 2021 and 2023 for osteoporosis and required extraction of three residual roots in the anterior left maxilla. Both patients subsequently developed clinical signs of MRONJ, including persistent mucosal defects, purulent discharge, and exposed jawbone, necessitating surgical referral. The first patient was successfully managed with a comprehensive surgical protocol analogous to the one described in this study, wherein sequestrectomy was performed in place of tooth extraction, while the second patient required hospitalization for more extensive surgical intervention. These contrasting outcomes further reinforce the hypothesis that rigorous adherence to a structured preventive protocol can significantly mitigate the risk of MRONJ, even among the most vulnerable populations.

A key component of our protocol was the application of CGF, which was used in all cases except for three patients who declined. CGF, a second-generation platelet concentrate, contains growth factors such as PDGF, TGF-β, and VEGF, which are known to promote angiogenesis, tissue regeneration, and wound healing [23]. Its application in extraction sockets may counteract the anti-angiogenic and anti-osteoclastic effects of ARAs, thereby facilitating healing in a compromised bony environment [24]. The high rate of complete mucosal healing at 90 days—despite delayed healing in some cases—suggests that CGF may play a critical role in supporting soft and hard tissue recovery.

Another important observation was the delayed mucosal healing in Group D (cancer patients) at the 10-day follow-up, with no sites showing complete healing. This may be attributed to the more potent and frequent dosing of ARAs in this group, as well as potential systemic immunosuppression or poor nutritional status [25–27]. However, by day 90, all sites had healed completely, indicating that while healing may be slower in cancer patients, it is still achievable with appropriate management. This delayed but eventual healing underscores the importance of extended follow-up in high-risk populations. It is well-established that ARAs can negatively affect osteoclast function and bone remodeling [7, 28], in addition to possessing anti-angiogenic effects that diminish vascular supply and heighten the risk of MRONJ [29, 30]. Consequently, by limiting bone trauma and preserving vascular integrity during surgery, we are able to mitigate the risk of MRONJ to a certain extent.

The fact that most extraction sites (74.60%) could not be tightly sutured did not appear to compromise healing outcomes [31, 32]. This challenges the conventional emphasis on primary closure in MRONJ-prone patients and suggests that secondary intention healing, when supported by CGF and local antimicrobials, can be equally effective. This is clinically significant, as it may reduce operative time and complexity, particularly in cases with significant bone loss or anatomic constraints.

Preoperative and postoperative CBCT imaging identified increased bone density at extraction sites in 26 and 31 patients, respectively. While such sclerosis may reflect the pharmacological effect of ARAs, it did not impede successful healing in any case. Notably, postoperative increases in bone density were observed in certain patients without any progression to MRONJ, underscoring that radiographic changes alone should not be conflated with pathological necrosis and reaffirming that clinical symptoms remain the cornerstone of MRONJ diagnosis. The presence of osteosclerosis in high-risk individuals does, however, raise the question of whether preoperative biopsy could help rule out subclinical MRONJ. Although biopsy was not performed in this study to avoid additional trauma, assessing its diagnostic utility in carefully selected cases represents a valuable direction for future research.

The comprehensive protocol also emphasized systemic and local infection control. All patients received preoperative antibiotics and postoperative mouthwash, which likely reduced the bacterial load and minimized the risk of secondary infection—a known trigger for MRONJ [13, 33]. The use of different mouthwashes (povidone-iodine, chlorhexidine, tinidazole) allowed for tailored regimens based on patient tolerance and clinical context, further enhancing compliance and effectiveness.

It is also worth noting that the majority of patients were able to resume ARAs within one month postoperatively without developing MRONJ. This supports the safety of brief drug holidays and timely resumption of essential therapy, which is crucial for managing underlying conditions such as osteoporosis or bone metastases. The presence of osteosclerosis in high-risk individuals does, however, raise the question of whether preoperative biopsy could help rule out subclinical MRONJ. Although biopsy was not performed in this study to avoid additional trauma, assessing its diagnostic utility in carefully selected cases represents a valuable direction for future research [34].

This study has several limitations. First, the absence of a control group restricts direct comparative assessment of the comprehensive protocol’s efficacy relative to conventional extraction approaches. Second, the single-center design may limit the generalizability of our findings. Third, the complete absence of MRONJ cases in this cohort prevents any meaningful analysis of specific risk factors associated with its development. Fourth, while the 90-day follow-up period aligns with AAOMS guidelines, it remains insufficient to identify potential late-onset MRONJ cases. A 90-day short-term observation can only confirm the absence of MRONJ in the immediate post-extraction period; determining whether MRONJ develops later requires longer-term follow-up to safeguard oral health. Our study is continuing with extended tracking and follow-up. Patients are instructed to contact us promptly for further management if they experience intraoral pain, erythema, swelling, or purulent discharge. Despite these limitations, the prospective design, rigorously standardized protocol, and high patient adherence collectively enhance the internal validity of our results. Future research should prioritize large-sample, multicenter randomized controlled trials to validate these findings, elucidate the individual contribution of each protocol component, and evaluate long-term outcomes beyond the 90-day timeframe.

## Conclusion

This prospective cohort study provides evidence that implementing an effective and rational treatment protocol during tooth extractions significantly benefits high-risk MRONJ patients. Adherence to such protocols minimizes the risk of postoperative infection, fosters improved healing of extraction sites, and, most importantly, maximizes the prevention of MRONJ. These findings underscore the importance of careful procedural planning and protocol-driven care in reducing adverse outcomes among vulnerable patient populations.

## Data Availability

Data used within this manuscript are available from the corresponding author on reasonable request.

## References

[1] King R, Tanna N, Patel V. Medication-related osteonecrosis of the jaw unrelated to bisphosphonates and denosumab-a review. Oral Surg Oral Med Oral Pathol Oral Radiol. 2019;127(4):289–99. 30713092 10.1016/j.oooo.2018.11.012

[2] Russell RGG. Bisphosphonates: the first 40 years. Bone. 2011;49(1). 10.1016/j.bone.2011.04.022 21555003

[3] Suryani IR, Shujaat S, Ivković U, Coucke W, Coropciuc R, Jacobs R. Risk of healing impairment following tooth extraction in patients administered with antiresorptive and non-antiresorptive polypharmacy. J Stomatol Oral Maxillofac Surg. 2024;125(2):101645. 37748709 10.1016/j.jormas.2023.101645

[4] Cuozzo A, Iorio-Siciliano V, Vaia E, Mauriello L, Blasi A, Ramaglia L. Incidence and risk factors associated to Medication-Related osteo necrosis of the jaw (MRONJ) in patients with osteoporosis after tooth extractions. A 12-months observational cohort study. J Stomatol Oral Maxillofac Surg. 2022;123(6):616–21. 35609780 10.1016/j.jormas.2022.03.020

[5] Suyama K, Otsuru M, Nakamura N, Morishita K, Miyoshi T, Omori K, et al. Bone resection methods in medication-related osteonecrosis of the jaw in the mandible: an investigation of 206 patients undergoing surgical treatment. J Dent Sci. 2024;19(3):1758–69. 39035329 10.1016/j.jds.2023.10.007 PMC11259631

[6] Sakamoto Y, Sawada S, Kojima Y. Medication-related osteonecrosis of the jaw without osteolysis on computed tomography: a retrospective and observational study. Sci Rep. 2023;13(1):12890. 37558709 10.1038/s41598-023-39755-6 PMC10412630

[7] Dipalma G, Inchingolo AM, Malcangi G, Ferrara I, Viapiano F, Netti A et al. Sixty-Month follow up of clinical MRONJ cases treated with CGF and piezosurgery. Bioeng (Basel). 2023;10(7). 10.3390/bioengineering10070863 PMC10376556 37508890

[8] Shimizu E, Tamasi J, Partridge NC. Alendronate affects osteoblast functions by crosstalk through EphrinB1-EphB. J Dent Res. 2012;91(3):268–74. 22180568 10.1177/0022034511432170 PMC3275334

[9] David P, Nguyen H, Barbier A, Baron R. The bisphosphonate tiludronate is a potent inhibitor of the osteoclast vacuolar H(+)-ATPase. J Bone Min Res. 1996;11(10):1498–507. 10.1002/jbmr.5650111017 8889850

[10] Coropciuc R, Coopman R, Garip M, Gielen E, Politis C, Van den Wyngaert T, et al. Risk of medication-related osteonecrosis of the jaw after dental extractions in patients receiving antiresorptive agents - A retrospective study of 240 patients. Bone. 2023;170:116722. 36858337 10.1016/j.bone.2023.116722

[11] Moon C, Kim H, Park JH, Park W, Kim HJ, Jung Y-S, et al. High-dose denosumab (Xgeva^®^) associated Medication-Related osteonecrosis of the jaws (MRONJ): incidence and clinical characteristics in a retrospective analysis of 1278 patients. Support Care Cancer. 2024;32(12):774. 39499349 10.1007/s00520-024-08974-6

[12] D’Agostino S, Valentini G, Dolci M, Ferrara E. Potential relationship between poor oral hygiene and MRONJ: an observational retrospective study. Int J Environ Res Public Health. 2023;20(7). 10.3390/ijerph20075402 PMC10094150 37048016

[13] Kwon Y-D, Jo H, Kim J-E, Ohe J-Y. A clinical retrospective study of implant as a risk factor for medication-related osteonecrosis of the jaw: surgery vs loading? Maxillofac Plast Reconstr Surg. 2023;45(1):31. 37707716 10.1186/s40902-023-00398-2 PMC10501104

[14] Arya R, Miles E, Sproat C, Patel D, Patel V. An institutional protocol including socket alveoplasty and primary closure following dental extractions for patients with an elevated risk of developing medication-related osteonecrosis of the jaw. Br Dent J. 2024;237(8):645–51. 39455783 10.1038/s41415-024-7968-5

[15] Liu C, Xiong Y-T, Zhu T, Liu W, Tang W, Zeng W. Management of tooth extraction in patients taking antiresorptive drugs: an evidence mapping review and meta-analysis. J Clin Med. 2022;12(1). 10.3390/jcm12010239 PMC9821631 36615038

[16] Ruggiero SL, Dodson TB, Aghaloo T, Carlson ER, Ward BB, Kademani D. American association of oral and maxillofacial surgeons’ position paper on Medication-Related osteonecrosis of the Jaws-2022 update. J Oral Maxillofac Surg. 2022;80(5):920–43. 35300956 10.1016/j.joms.2022.02.008

[17] Jelin-Uhlig S, Weigel M, Ott B, Imirzalioglu C, Howaldt H-P, Böttger S et al. Bisphosphonate-related osteonecrosis of the jaw and oral microbiome: clinical risk factors, pathophysiology and treatment options. Int J Mol Sci. 2024;25(15). 10.3390/ijms25158053 PMC11311822 39125621

[18] McGowan K, Ware RS, Acton C, Ivanovski S, Johnson NW. Both non-surgical dental treatment and extractions increase the risk of medication-related osteonecrosis of the jaw: case-control study. Clin Oral Investig. 2019;23(11):3967–75. 30747305 10.1007/s00784-019-02828-w

[19] McGowan K, McGowan T, Ivanovski S. Risk factors for medication-related osteonecrosis of the jaws: A systematic review. Oral Dis. 2018;24(4):527–36. 28656643 10.1111/odi.12708

[20] Ruggiero SL, Fantasia J, Carlson E. Bisphosphonate-related osteonecrosis of the jaw: background and guidelines for diagnosis, staging and management. Oral Surg Oral Med Oral Pathol Oral Radiol Endod. 2006;102(4):433–41. 16997108 10.1016/j.tripleo.2006.06.004

[21] Beninati F, Pruneti R, Ficarra G. Bisphosphonate-related osteonecrosis of the jaws (Bronj). Med Oral Patol Oral Cir Bucal. 2013;18(5):e752–8. 23722119 10.4317/medoral.18076 PMC3790648

[22] Tardast A, Sjöman R, Løes S, Abtahi J. Bisphosphonate associated osteomyelitis of the jaw in patients with bony exposure: prevention, a new way of thinking. J Appl Oral Sci. 2015;23(3):310–4. 26221926 10.1590/1678-775720140506 PMC4510666

[23] Besi E, Pitros P. The role of leukocyte and platelet-rich fibrin in the prevention of medication-related osteonecrosis of the jaw, in patients requiring dental extractions: an observational study. Oral Maxillofac Surg. 2024;28(2):785–93. 38182917 10.1007/s10006-023-01204-z

[24] Parise GK, Costa BN, Nogueira ML, Sassi LM, Schussel JL. Efficacy of fibrin-rich platelets and leukocytes (L-PRF) in tissue repair in surgical oral procedures in patients using Zoledronic acid-case-control study. Oral Maxillofac Surg. 2023;27(3):507–12. 35739366 10.1007/s10006-022-01094-7 PMC9225877

[25] Uyanne J, Calhoun CC, Le AD. Antiresorptive drug-related osteonecrosis of the jaw. Dent Clin North Am. 2014;58(2):369–84. 24655528 10.1016/j.cden.2013.12.006

[26] Chang J, Hakam AE, McCauley LK. Current Understanding of the pathophysiology of osteonecrosis of the jaw. Curr Osteoporos Rep. 2018;16(5):584–95. 30155844 10.1007/s11914-018-0474-4

[27] Ng TL, Tu MM, Ibrahim MFK, Basulaiman B, McGee SF, Srikanthan A, et al. Long-term impact of bone-modifying agents for the treatment of bone metastases: a systematic review. Support Care Cancer. 2021;29(2):925–43. 32535678 10.1007/s00520-020-05556-0

[28] Seluki R, Seluki M, Vaitkeviciene I, Jagelaviciene E. Comparison of the effectiveness of Conservative and surgical treatment of Medication-Related osteonecrosis of the jaw: a systematic review. J Oral Maxillofac Res. 2023;14(4):e1. 38222882 10.5037/jomr.2023.14401 PMC10783881

[29] Zhou J, Ma X, Wang T, Zhai S. Comparative efficacy of bisphosphonates in short-term fracture prevention for primary osteoporosis: a systematic review with network meta-analyses. Osteoporos Int. 2016;27(11):3289–300. 27273112 10.1007/s00198-016-3654-z

[30] Tang X, Zhang Q, Shi S, Yen Y, Li X, Zhang Y et al. Bisphosphonates suppress insulin-like growth factor 1-induced angiogenesis via the HIF-1alpha/VEGF signaling pathways in human breast cancer cells. Int J Cancer. 2010;126(1). 10.1002/ijc.24710 PMC2784023 19569175

[31] Spanou A, Nelson K, Ermer MA, Steybe D, Poxleitner P, Voss PJ. Primary wound closure and perioperative antibiotic therapy for prevention of bisphosphonate-related osteonecrosis of the jaw after tooth extraction. Quintessence Int. 2020;51(3):220–8. 32020132 10.3290/j.qi.a43949

[32] Otto S, Tröltzsch M, Jambrovic V, Panya S, Probst F, Ristow O, et al. Tooth extraction in patients receiving oral or intravenous bisphosphonate administration: A trigger for BRONJ development? J Craniomaxillofac Surg. 2015;43(6):847–54. 25958767 10.1016/j.jcms.2015.03.039

[33] He L, Sun X, Liu Z, Qiu Y, Niu Y. Pathogenesis and multidisciplinary management of medication-related osteonecrosis of the jaw. Int J Oral Sci. 2020;12(1):30. 33087699 10.1038/s41368-020-00093-2 PMC7578793

[34] Jacob G, Thomas, Aviv Ouanounou. Medication-related osteonecrosis of the jaw: a narrative review of risk factors, diagnosis, and management. Front Oral Maxillofacial Med. 2022;5.

